# Using allogenous structural bone graft for uncontained tibial bone defects ≥ 10 mm in depth in primary total knee arthroplasty

**DOI:** 10.1186/s12891-022-05491-7

**Published:** 2022-06-02

**Authors:** Dai Iwase, Yukie Metoki, Yasuaki Kusumoto, Jun Aikawa, Kensuke Fukushima, Shotaro Takano, Manabu Mukai, Kentaro Uchida, Gen Inoue, Masashi Takaso

**Affiliations:** 1 Department of Orthopedic Surgery, Kitasato University School of Medicine, 1-15-1 Kitasato, Minami-ku, Sagamihara City, Kanagawa, 252-0374 Japan; 2 Department of Physical Therapy, Fukushima Medical University School of Health Sciences, 10-6 Sakaemachi, Fukushima, 960-8516 Japan

**Keywords:** Tibial bone defect, Radiography, Primary total knee arthroplasty, Bone graft, Allogenous structural bone graft

## Abstract

**Background:**

In primary total knee arthroplasty (TKA), tibial bone defects ≥ 10 mm in depth often become uncontained defects, a condition most surgeons find challenging to treat. Although the allogenous bone graft is a useful method, complications such as infection and nonunion are likely to occur. There are several reports on the use of allogenous bone graft in revision TKA; however, few studies have investigated its use in primary TKA. We performed primary TKA using the allogenous bone graft as a structural bone graft to treat uncontained defects ≥ 10 mm in depth. This study aimed to assess the clinical and radiographical results after primary TKA with allogenous structural bone graft (ASBG).

**Methods:**

Seventeen patients (mean age, 69.2 years) with a follow-up period of at least 7 years, were retrospectively reviewed. All cases had been treated for medial bone defects using the ipsilateral medial tibial allogenous bone. Clinical evaluation included the assessment of the knee and function scores and knee angle, and the hip-knee-ankle (HKA) angle, bone union, and radiolucent line (RL) were assessed radiologically.

**Results:**

The mean depth of the medial tibial defects after tibia cutting was 16.8 mm. Nonunion occurred in one case, and RL occurred in another. We observed a significant difference when the preoperative knee score and HKA angle of patients was compared with that at 1 year postoperatively and the final evaluation. No major complications were observed.

**Conclusion:**

The ASBG technique produced favorable surgical outcomes and may be an acceptable procedure for managing uncontained tibial bone defects ≥ 10 mm in depth in primary TKA.

## Background

Primary total knee arthroplasty (TKA) produces good long-term results. However, we sometimes encounter cases with medial tibial uncontained bone defects, which require some ingenuity. Generally, uncontained bone defects < 5 mm in depth are filled with bone cement, while defects of 5–10 mm in depth are treated with bone graft, and defects ≥ 10 mm in depth are treated using metal augmentation [1]. When the bone defect is ≥ 10 mm in-depth, the management can be challenging. The use of metal augmentation for uncontained defects ≥ 10 mm in depth creates further bone loss and can make future revision surgery difficult. Although there have been a few reports of primary TKA with autologous bone graft for uncontained tibial bone defects ≥ 10 mm in depth [2, 3], it is difficult to obtain a bone block ≥ 10 mm thick from the resected femoral or tibial bone. An allogenous bone graft is useful because it is freely shaped, is usually available in sufficient quantity, and is usable as a structural bone graft. However, most of the reports using allogenous bone graft have been for revision TKA or primary and revision TKA, not for primary TKA alone. Therefore, in the present study, we aimed to assess clinical and radiographical results following primary TKA with allogenous structural bone graft (ASBG).

## Methods

We performed primary TKA using medial tibial allogenous bone as a structural bone graft for uncontained defects ≥ 10 mm in depth. Further, we assessed the clinical and radiographical results after primary TKA with ASBG for uncontained tibial bone defects ≥ 10 mm in depth.

For this study, we retrospectively enrolled 35 patients who underwent TKA with allogenous bone graft at a single institution between January 2008 and December 2014. The study included patients with a record of primary TKA for ≥ 10 mm deep uncontained medial bone defects following proximal tibial cutting, the use of the allogenous bone of ipsilateral medial condyle of tibia, and a clinical and radiological follow-up period of at least 7 years. Patients with valgus knees and patients who underwent revision TKA were excluded. Of the 35 patients, 21 underwent primary TKA using ASBG. Of these, three patients used the allogenous bone of the femoral head for contained defect and one patient relocated and could not be followed up for more than 7 years. Finally, 17 patients (17 knees) met all the inclusion criteria. Fifteen women and two men constituted the study sample, and the mean age at surgery was 69.2 years (range, 50–83 years). The diagnoses were osteoarthritis (*n* = 11) and rheumatoid arthritis (*n* = 6). The mean follow-up duration was 96.4 months (range, 84–150 months). The prostheses included 13 Scorpio NRG® (Stryker Orthopaedics, NJ, USA), three Scorpio TS® (Stryker Orthopaedics, NJ, USA), and one Triathlon® (Stryker Orthopaedics, NJ, USA).

### Surgical procedures

All TKAs were performed using a measured resection technique with a midline incision and a medial parapatellar arthrotomy. Balancing was performed by manual evaluation with femoral and tibial trial implants and articulating tibial inserts. In addition, all implants were cement-fixed.

**Fig. 1:**
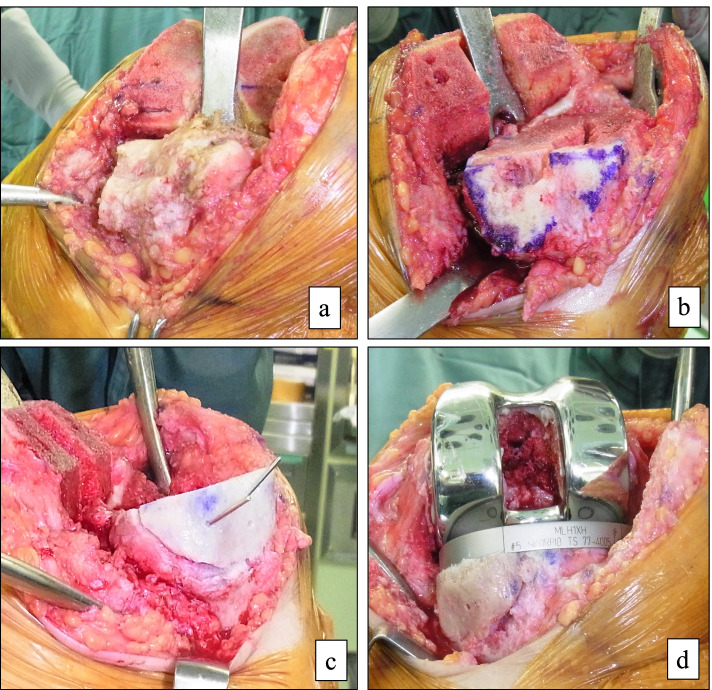
Bone-grafting technique for defect with no anteroposterior rim (**a**) The bone defect before tibial osteotomy. (**b**) The host bone was cut obliquely to create a slope without anteroposterior rim after tibial osteotomy. (**c**) The allogenous structural bone graft (ASBG) temporarily fixed to the host bone with Kirshner wires. (**d**) Kirshner wires were removed after cementing the tibial implant and ASBG press-fitted to the host bone

**Fig. 2:**
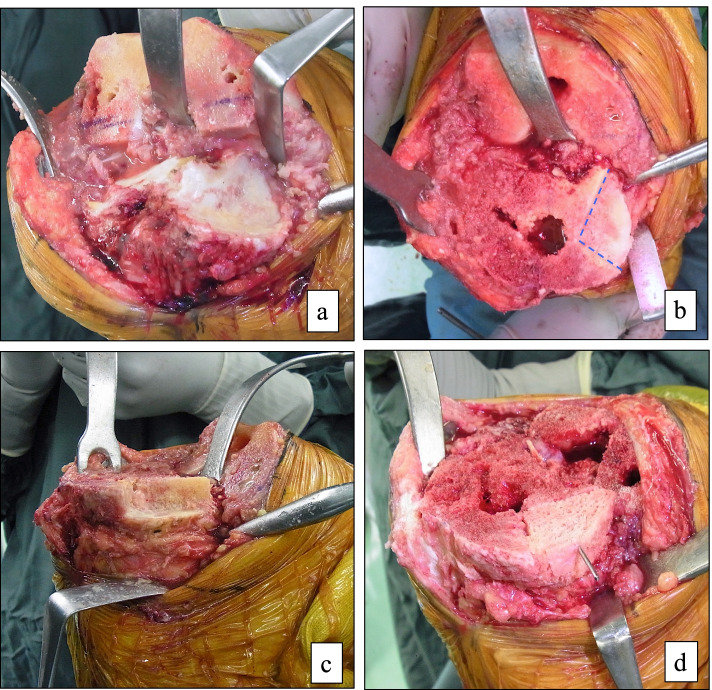
Bone-grafting technique for defect with residual anteroposterior rim (**a**) The bone defect before tibial osteotomy. (**b** and **c**) The host bone was cut to create a basket shape with the residual anteroposterior rims after tibial osteotomy; the dotted line is the boundary of the defect. (**d**) The allogenous structural bone graft (ASBG) was temporarily fixed to the host bone with Kirshner wire

The tibial bone was cut to a depth of 8 to 10 mm from the articular surface of the lateral tibial condyle. No more than 10 mm of the tibia was removed because additional removal would have resulted in an unacceptably small tibial surface and a lower joint line. The sclerotic surface of the medial tibial bone defect was refreshed to expose a flat cancellous bony surface. In the absence of anteroposterior cortical rims, the host bone was cut obliquely beneath the defect, resulting in a slope surface, to preserve as much bone as possible (Fig. 1a, b). Conversely, in the presence of anteroposterior cortical rims, the host bone was cut to form a basket shape (Fig. 2a-c). The bone defect was measured using a sterilized ruler (depth, anteroposterior width, and mediolateral width). For ASBG, we used the proximal medial tibial allogenous bone on the same side. The allogenous bone was carefully structured to fit the bone defect and reconstruct the missing rim. A precision fit of the bone graft to the defect was essential to prevent cement from entering the interface. Kirschner wires (1.5 mm) were used to provisionally stabilize the allogenous bone to the host bone (Fig. 1c, Fig. 2d) and were removed after cementing the tibial implant (Fig. 1d). We used tibial extension stems with using ASBG. The length of the extension stem was greater than the depth of the bone defect.

Postoperative rehabilitation was similar to that prescribed for patients without bone grafting. The day after surgery, patients started quadriceps femoris strengthening exercises, with no restriction placed on weight-bearing. The drainage catheter was removed on the second postoperative day, and the patients commenced with passive and active joint exercise.

### Radiological assessments

**Fig. 3:**
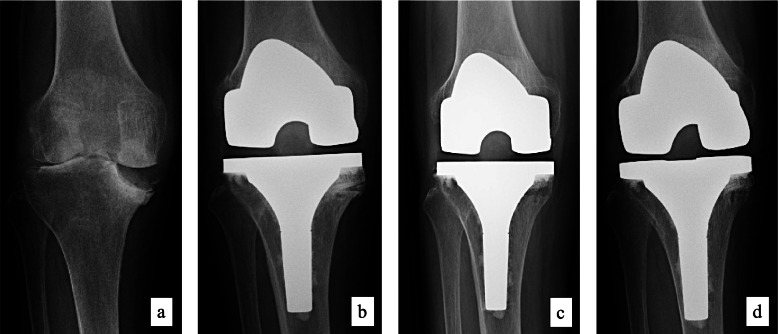
Pre- and postoperative follow-up radiographs of a 67-year-old woman patient (case 14) (**a)** Preoperatively. (**b)** Immediately after surgery. (**c)** Three months postoperatively. (**d)** Final follow-up

The hip-knee-ankle (HKA) angle was measured preoperatively, 1 year postoperatively, and at the final follow-up using weight-bearing anteroposterior radiographs. Bone union and radiolucent line (RL) were assessed immediately after surgery, 3 and 6 months after surgery, and at the final follow-up using non-weight bearing anteroposterior and lateral radiographs (Fig. 3). RL > 2 mm was defined as positive. Two orthopedic surgeons who were members of the Japanese Orthopedic Association evaluated the radiographs.

### Clinical assessments

The knee and function scores of the Knee Society clinical rating system [4] and the knee angle were evaluated preoperatively, 1 year postoperatively, and at the final follow-up, and complications were identified from the medical records.

### Statistical analysis

The normality of radiological and clinical parameters was confirmed using the Shapiro–Wilk test. The postoperative course of these parameters was examined using repeated-measures one-way analysis of variance and multiple comparison test (Bonferroni method). The survival rate of avoided revision in the postoperative course was also calculated. Statistical analyses were performed using IBM SPSS Statistics for Windows, ver. 27 (IBM Corp., Armonk, Tokyo, Japan). A *p-*value of ≤ 0.01 implied statistical significance.

## Results

**Fig. 4:**
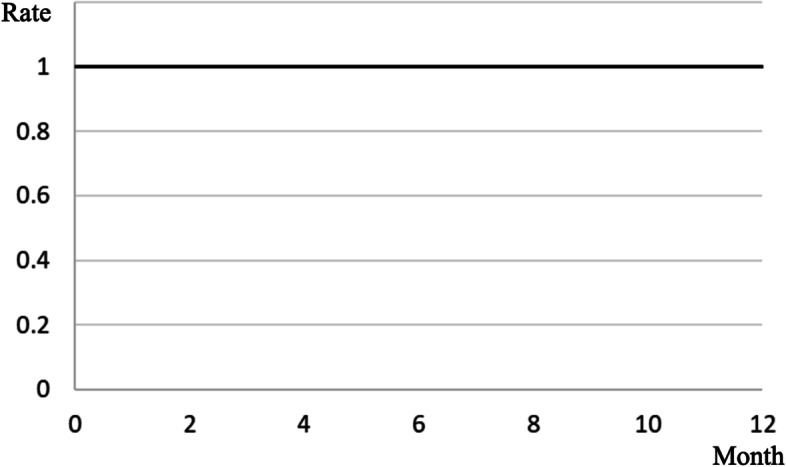
Survival rate of avoided revision

Patient demographic data is summarized in Table 1. The mean size of the medial tibial defects after tibia cutting was 16.8 mm (range, 12–30 mm) in depth, 34.4 mm (range, 24–40 mm) in anteroposterior width, and 25.4 mm (range, 17–35 mm) in mediolateral width. Of the 17 cases, nonunion was observed in one patient (case 3), who showed resorption of the transplanted bone, and the presence of RL was observed in one patient (case 13). Bone union was confirmed in an average of 4.9 months (Table 2). The results of the repeated measures one-way analysis of variance showed a significant improvement in the HKA and the knee score at 1 year postoperatively and at the final evaluation compared with the preoperative assessment. Preoperative function score, knee flexion, and extension did not differ significantly from the assessment 1 year postoperatively and at the final follow-up, although they showed a tendency to improve (Table 3). No major complications, such as transmission of infection, dislocation of graft bone, or loosening of the prosthesis, were observed. Therefore, there were no cases of revision TKA, and the survival rate of avoided revision was 100% (Fig. 4).

**Table 1:** Baseline characteristics of patients included in this study

Case	Age (years)	Sex	Diagnosis	Defect measurements after tibia cutting (mm)		
				AP width	ML width	Depth
1	65	M	RA	40	20	12
2	57	F	OA	24	17	17
3	50	F	RA	31	28	23
4	78	F	OA	36	27	18
5	74	F	OA	45	35	13
6	81	M	OA	40	30	18
7	79	F	OA	20	22	17
8	62	F	RA	35	30	15
9	81	F	OA	39	30	20
10	72	F	OA	40	25	10
11	83	F	OA	35	20	14
12	62	F	OA	32	29	27
13	68	F	RA	30	25	15
14	67	F	RA	37	23	12
15	50	F	RA	25	23	30
16	76	F	OA	35	28	14
17	72	F	OA	40	20	10

*M* Male, *F* Female, *OA* Osteoarthritis, *RA* Rheumatoid arthritis, *AP* Anteroposterior, *ML* Mediolateral

**Table 2:** Radiological results after surgery

HKA angle			Bone union	Union time (months)	RL
Preoperatively	1 year postoperatively	Final follow-up			
21.9 ± 9.1	2.7 ± 2.7*	3.2 ± 3.0**	16/17 (94.1%)	4.9 ± 2.4	1/17 (5.9%)

*HKA* hip-knee-ankle, *RL* radiolucent line

Means ± standard deviation;

^*^ denotes *p* < 0.01, Preoperatively vs. 1 year postoperatively;

^**^ denotes *p* < 0.01, Preoperatively vs. final follow-up

**Table 3:** Clinical results after surgery

	Preoperatively	1 year postoperatively	Final follow-up
Knee score	15.4 ± 10.0	92.0 ± 8.2*	91.7 ± 8.5**
Function score	35.0 ± 23.5	54.7 ± 29.1	52.7 ± 32.8
Knee flexion	106.2 ± 17.3	112.4 ± 12.5	113.2 ± 15.1
Knee extension	-10.0 ± 11.6	-4.4 ± 9.0	-2.4 ± 8.7

Means ± standard deviation;

^*^ denotes *p* < 0.01, Preoperatively vs. 1 year postoperatively;

^**^ denotes *p* < 0.01, Preoperatively vs. final follow-up

### Case presentation

**Fig. 5:**
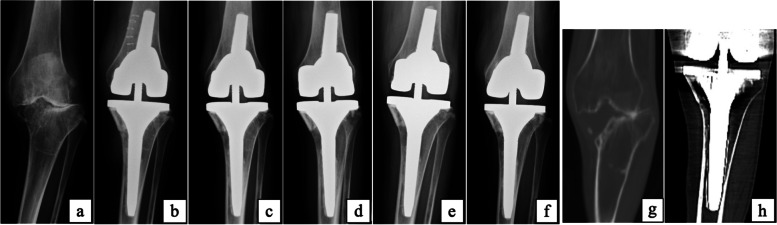
Pre- and postoperative follow-up radiographs and computed tomography (CT) scan (**a**) Preoperatively. (**b**) Immediately after surgery. (**c**) 6 months after surgery. (**d**) 3 years after surgery. (**e**) 7 years after surgery. (**f**) Final follow-up (11.5 years). (**g**) CT scan preoperatively. (**h**) CT scan at 3 years after surgery

The patient was a 50-year-old woman with rheumatoid arthritis. The size of the medial tibial defect after tibia cutting was 30 mm in depth, 25 mm in anteroposterior width, and 23 mm in mediolateral width (Fig. 5a, g). Scorpio TS TKA with ASBG was performed and follow-up period was 11.5 years. Bone union was confirmed at 6 months after surgery, and no RL or loosening was observed until final follow-up. HKA improved from 19.5° preoperatively to 3° at the final follow-up (Fig. 5b-f, h). The knee score improved from 8 preoperatively to 87 points. The functional score was 0 points preoperatively, and it did not change even at the final follow-up.

## Discussion

In this study, primary TKA using ASBG was performed on 17 knees with uncontained defects ≥ 10 mm in depth. Bone union was achieved in 94% of the knees at 96.4 months follow-up. In addition, knee score and HKA angle showed sustained improvement until the final follow-up when compared to the preoperative scores, and there were no major complications.

There are two types of bone defects in the proximal medial tibia: (i) contained defects showing that preservation of the cortical rim can support the tibial prosthesis, and (ii) uncontained defects without the cortical rim and cannot support the prosthesis. Uncontained defects are often observed in patients with severe varus deformity [5]. Dorr et al. noted that prosthesis loosening and postoperative knee deformities are likely to occur in uncontained defects; they found that repairing the defect with bone cement was not adequate and promoted the use of bone graft as a useful treatment method [6]. Since then, although several authors reported satisfactory results following an autologous bone graft in primary TKA, most reports were of uncontained defects ≥ 5 mm in depth [6–8]. Generally, uncontained bone defects ≥ 10 mm in depth are treated using metal augmentation [1], and some reports [9, 10] recommend TKA with metal augmentation as a useful method. However, the use of additional metal augmentation requires further bone cutting, including the cortical rim and can make future revision TKA difficult. On the other hand, there are only a few studies on treatment with autologous bone grafts for uncontained tibial bone defects ≥ 10 mm in depth [2, 3], and no reports on primary TKA with allogenous bone graft.

The advantage of a bone graft is that once incorporated, it becomes a part of the proximal tibia and is, therefore, durable. The load transfer to the underlying tibia is more physiologic if the bone grafts are successful compared to using metal custom components without a bone cement composite. Moreover, structural bone grafts are superior to morselized bone grafts in the initial fixation and are reported to yield good results in primary TKA [5, 6, 11]. However, with the combined use of autologous bone graft from a resected femoral or tibial bone, it is difficult to obtain a bone block ≥ 10 mm thick and reconstruct the rim, including the cortex [3]. Allogenous bone grafts can be freely sized and shaped, and it is possible to reconstruct a rim, including the cortex, with almost the same shape as bones used from the same site. In the present study, we were able to reconstruct the rim for any form of defect using ASBG.

Several authors [11–13] have associated allogenous bone graft with an increased risk of transmission of infection, nonunion, and resorption. However, almost all reports are on revision TKA with allogenous bone grafts. As primary TKA is less complicated with a shorter operation time than revision TKA, we considered that the risk of infection might be lower. In this study, we observed no infection and achieved bone union in 94% of the cases.

Generally, the grafted bone is fixed with screws. Screw support can achieve rigid initial fixation, and the rigidity also helps to maintain long-term alignment [6, 8]. On the other hand, insertion of multiple screws may lead to fragmentation of the grafted bone, resulting in early failure of knee replacement [5]. As a device that does not use screw fixing, Chon et al. [2] used the oblique structural peg bone, while Yoon et al. [7] applied a cement to the medial surface of the tibia to fix the grafted bone. We temporarily fixed the allogenous grafted bone with Kirschner wires and withdrew them after fixation of the tibial component with extension stems. Extension stems may result in a future loss of bone stock when performing revision surgery. In addition, Scott et al. [14] reported that extension stems might have disadvantages, including stress shielding along the length of the stem with associated reduction in bone density and a theoretical risk of subsidence and loosening, peri-prosthetic fracture, and end-of-stem pain. However, Rawlinson et al. [15] used cadaver knees to biochemically confirm that using a tibial extension stem reduced bone stress and limited micromotion between the metal wedge and surrounding bone, and hence, recommended using a tibial extension stem. Baek et al. [9] reported that bone grafting could not provide sufficient stability for severe bone defects ≥ 10 mm in depth in primary TKA; thus, requiring an extension stem, particularly for uncontained bone defects where fixation of the grafted bone was technically difficult. We did not fix the grafted bone because we considered that we could obtain the initial strength with a combination of the tibial extension stem and performing ASBG, including the rim; we did not observe dislocation of the ASBG and/or implant.

We acknowledge that the present study had certain limitations. First, this was a single-center study, and the study sample (17 cases) was small. Although Chon et al. [2] and Sugita et al. [3] reported 40 cases and 44 cases, respectively, in the literature using autologous bone graft for bone defects ≥ 10 mm, in this study, only 21 (4%) of 525 primary TKAs from January 2004 to December 2014 had a medial tibial defect ≥ 10 mm in depth; thus, such defects can be considered rare. Second, we have not been able to compare this with other methods, such as autologous bone graft or metal augmentation. Sugita et al. [3] reported 14% non-progressive RL with autologous bone graft, Beak et al. [9] and Tsukada et al. [16] reported non-progressive RL in 10% and 33%, respectively, combined with metal augmentation. In this study, nonunion and presence of RL were 5.9% each, which did not differ from the other methods. Despite the absence of a control group, we believe that our findings are adequate; thus, important conclusions can be drawn from them.

## Conclusions

The ASBG technique for uncontained tibial bone defects ≥ 10 mm in depth in primary TKA provided favorable clinical and radiological outcomes, thereby confirming its use as an acceptable procedure.

## Data Availability

The datasets supporting the conclusions of this article are included within the article. The corresponding author can provide raw data on request.

## Abbreviations

TKA: Total knee arthroplasty
ASBG: Allogenous structural bone graft
IRB: Institutional Review Board
HKA: Hip-knee-ankle
RL: Radiolucent line

## References

[1] Vail TP, Lang JE, Sikes CV, Scott WN (2012). Surgical techniques and instrumentation in total knee arthroplasty. Insall & Scott surgery of the knee.

[2] Chon JG, Kang JW, Kim CU, Jeong U, Go J (2021). Treatment of 10-mm-deep or greater uncontained tibial bone defects in primary total knee reconstruction without metal augmentation: autologous oblique structural peg bone and cancellous chip bone grafting. Clin Orthop Surg.

[3] Sugita T, Aizawa T, Miyatake N, Sasaki A, Kamimura M, Takahashi A (2017). Preliminary results of managing large medial tibial defects in primary total knee arthroplasty: autogenous morcellised bone graft. Int Orthop.

[4] Insall JN, Dorr LD, Scott RD, Scott WN (1989). Rationale of the Knee Society clinical rating system. Clin Orthop Relat Res.

[5] Ahmed I, Logan M, Alipour F, Dashti H, Hadden WA (2008). Autogenous bone grafting of uncontained bony defects of tibia during total knee arthroplasty a 10-year follow up. J Arthroplasty.

[6] Dorr LD, Ranawat CS, Sculco TA, McKaskill B, Orisek BS (1986). Bone graft for Tibial defects in total knee arthroplasty. Clin Orthop Relat Res.

[7] Yoon JR, Seo IW, Shin YS (2017). Use of autogenous onlay bone graft for uncontained tibial bone defects in primary total knee arthroplasty. BMC Musculoskelet Disord.

[8] Hosaka K, Saito S, Oyama T, Fujimaki H, Cho E, Ishigaki K (2017). Union, knee alignment, and clinical outcomes of patients treated with autologous bone grafting for medial tibial defects in primary total knee arthroplasty. Orthopedics.

[9] Baek SW, Kim CW, Choi CH (2013). Management of tibial bony defect with metal block in primary total knee replacement arthroplasty. Knee Surg Relat Res.

[10] Lee JK, Choi CH (2011). Management of tibial bone defects with metal augmentation in primary total knee replacement: a minimum five-year review. J Bone Joint Surg Br.

[11] Mullaji AB, Padmanabhan V, Jindal G (2005). Total knee arthroplasty for profound varus deformity: technique and radiological results in 173 knees with varus of more than 20 degrees. J Arthroplasty.

[12] Bauman RD, Lewallen DG, Hanssen AD (2009). Limitations of structural allograft in revision total knee arthroplasty. Clin Orthop Relat Res.

[13] Dennis DA (2002). The structural allograft composite in revision total knee arthroplasty. J Arthroplasty.

[14] Scott CE, Biant LC (2012). The role of the design of tibial components and stems in knee replacement. J Bone Joint Surg Br.

[15] Rawlinson JJ, Closkey RF, Davis N, Wright TM, Windsor R (2008). Stemmed implants improve stability in augmented constrained condylar knees. Clin Orthop Relat Res.

[16] Tsukada S, Wakui M, Matsueda M (2013). Metal block augmentation for bone defects of the medial tibia during primary total knee arthroplasty. J Orthop Surg Res.

